# Supplementary material for: Patterns of Intron Gain and Loss in Fungi
Source: PLoS Biol. 2004 Nov 30;2(12):e422. doi: 10.1371/journal.pbio.0020422 (PMC532390; doi:10.1371/journal.pbio.0020422)
Supplement: Table S1 — Also available at http://genes.mit.edu/NielsenEtAl/. (4.3 MB ZIP). [file pbio.0020422.st001.zip › NielsenEtAl/html/728.html]

AN2438.1.NCU09745.1.MG06110.1.FG05298.1


```
 CLUSTAL W (1.82) Multiple Sequence Alignments - Introns Inserted


Sequence 1: NCU09745.1	343 aa
Sequence 2: MG06110.1	315 aa
Sequence 3: FG05298.1	358 aa
Sequence 4: AN2438.1	287 aa
Alignment Length: 364 aa
Number Identitical Residues: 140 aa
Alignment Score (without introns) 7631


MG06110.1 	----------------------~------MG0FLGAIGRACSVVNLDPANDHTSYPCALD
NCU09745.1	---------------------M~TSPLPVQQ~FMGAIGRQCSVVNLDPANDHTNYPCALD
FG05298.1 	MPFAQLVLGSPGCGKSTYCDGI1QLTGQVHQ~FLGAIGRACSVVNLDPANDHTNYPAALD
AN2438.1  	----------------------~--------~----------------------------
          	                                                            

MG06110.1 	IRNLVTLEEIMGDDNLGPNGGILYAIEELEHNFEWLEDGLKELGD~DYILFDCPGQVELY
NCU09745.1	IRDLVTLEEIMADDKLGPNGGILYALEELENNMEWLENGLKELGE~DYVLFDCPGQVELY
FG05298.1 	IRSLIKLEEIMKDDKLGPNGGILYALEELEHNFEWLEEGLKEFSE~DYILFDCPGQVELY
AN2438.1  	----------MSEDQLGPNGGVLYALEELEENFDFLEEGLKELGE1DYIIFDCPGQVEIF
          	          * :*:******:***:****.*:::**:****:.: **::********::

MG06110.1 	THHNSLRNIFFKLQKLGYR0LVVVHLSDSICLTQPSLYISNLLLALRAMLQMDLSHVNVL
NCU09745.1	THHNSLRNIFYRLQKLGYR0LVVVHLSDCFCLTQPSLYISNVLLSLRAMLQMDLPHINVL
FG05298.1 	THHNSLRNIFYKLQKIGFR0LVSVHLSDSFCLTQPSLYVSNVLLSLRAMIQMDMPHINIL
AN2438.1  	THHSSLRNIFFKIQKMGYR0LIVLHLIDSYNLTLPSMYISSLILCLRAMLQMDLPHLNVL
          	***.******:::**:*:* *: :** *.  ** **:*:*.::*.****:***:.*:*:*

MG06110.1 	TKIDKVSSYDRLAFNLDFYTEVHDLSYLLPELEAENPSLRSEKFAKLNRAVANLIEDFGL
NCU09745.1	TKIDKISSYDPLPFNLDYYTEVQDLRYLMPSLDAESPALKKGKFTKLNEAVANMVEQFGL
FG05298.1 	SKIDKVADYDELPFNLDYYTDVDDLTYLTPHLETESPALRSEKFGKLNEAIANLIESYGL
AN2438.1  	TKIDNLSNYTSLPFNLDFYTEVQDLTYLLPHLEAESSRLSHEKFGALNNAIITLIEEFGL
          	:***:::.*  *.****:**:*.** ** * *::*.. *   **  **.*: .::*.:**

MG06110.1 	VRFEVLAVENKKSMMHLLRVLDRANGYVFGGAEGANDTVWQVAMRNEGSLMGVQDIQERW
NCU09745.1	VSFEVLAVENKKSMMHLLRVIDRASGYVFGGAEGTNDTVWQVAMRNESSLPDALDIQERW
FG05298.1 	VRYEVLAVENKKSMMHILRVIDRAGGYVFGSAEGANDTVWSVAMRNESSMLGVQDIQERW
AN2438.1  	VGFETLAVEDKKSMMNLLRAIDRASGYVFGPAEGANDSVWQVAVREGMGSMDIRDIQERW
          	* :*.****:*****::**.:***.***** ***:**:**.**:*:  .  .  ******

MG06110.1 	IDNKEAYDEMEQREWEEQVKAQEAMAEADAAAAE-EG-----DDDLMGGPGAR2---~--
NCU09745.1	IDSKEEYDEMERKEEEEQEKLRAEQARAAEEAGLGDGSVPGVAPQFTSGSGIR~VTL1SL
FG05298.1 	IDQKVEYDQMEREAEEEQARIQEEQAMEMEQSQPPPAPTGGMDPDFGDMTVPK~D--~SG
AN2438.1  	IDAKDEYDELERRQREEEIKNHQQAATYQAGNEDDDD-----DNDYEFGRRMP~VPD~SG
          	** *  **::*:.  **: : :   *                  :          .  : 

MG06110.1 	-------~---
NCU09745.1	VAAFTKY1SDL
FG05298.1 	IKVVRK-~---
AN2438.1  	VKVMRK-~---
          	     .
```
